# Comparison between 5-day decitabine and 7-day azacitidine for lower-risk myelodysplastic syndromes with poor prognostic features: a retrospective multicentre cohort study

**DOI:** 10.1038/s41598-019-56642-1

**Published:** 2020-01-08

**Authors:** Byung-Hyun Lee, Ka-Won Kang, Min Ji Jeon, Eun Sang Yu, Dae Sik Kim, Hojoon Choi, Se Ryeon Lee, Hwa Jung Sung, Byung Soo Kim, Chul Won Choi, Yong Park

**Affiliations:** 1 Department of Internal Medicine, Korea University College of Medicine, Anam Hospital, Seoul, Korea; 2 Department of Internal Medicine, Korea University College of Medicine, Guro Hospital, Seoul, Korea; 3 Department of Internal Medicine, Korea University College of Medicine, Ansan Hospital, Gyeonggi-do, Korea

**Keywords:** Myelodysplastic syndrome, Myelodysplastic syndrome

## Abstract

Numerous studies have analysed the clinical efficacies of hypomethylating agents (HMAs) in patients with myelodysplastic syndromes (MDS). However, reports that compare the two HMAs, decitabine and azacitidine, in patients with lower-risk (low and intermediate-1) MDS are limited. We compared 5-day decitabine and 7-day azacitidine regimens in terms of treatment responses, survival outcomes, and adverse events in patients with lower-risk MDS with poor prognostic features. The overall response rates (ORRs) were 67.2% and 44.0% in the patients treated with decitabine and azacitidine, respectively (*P* = 0.014). While the median progression-free survival (PFS) was significantly better in the patients treated with decitabine than in those treated with azacitidine (*P* = 0.019), no significant differences in event-free and overall survival rates were observed between the two groups. Multivariate analysis revealed that compared with azacitidine treatment, decitabine treatment is significantly associated with a higher ORR (*P* = 0.026) and longer PFS (*P* = 0.037). No significant differences were observed in the incidence of grade 3 or higher haematologic adverse events in response to the two HMAs. In conclusion, in lower-risk MDS, especially with poor prognostic features, ORR and PFS were significantly better with 5-day decitabine treatment than with 7-day azacitidine treatment, with comparable safety.

## Introduction

Myelodysplastic syndromes (MDS) are clonal haematologic disorders characterised by ineffective and dysplastic haematopoiesis that causes cytopenia, leading to the development of acute leukaemia^1,2^. Previously, MDS was generally divided into lower-risk (low and intermediate-1) and higher-risk (intermediate-2 and higher) categories on the basis of the International Prognostic Scoring System (IPSS)^3^, and treatment decisions were usually made based on the risk group. The treatment for higher-risk MDS aims to modify the natural course of the disease using hypomethylating agents (HMAs), chemotherapy, or stem cell transplantation, whereas the treatment for lower-risk MDS aims to improve cytopenia, reduce transfusion requirements, and provide the best supportive care^4^. However, lower-risk MDS patients with poor prognostic features were known to be associated with progression to acute myeloid leukaemia or severe cytopenia^4^. Hence, more active treatments are needed for this group of patients.

Currently, two types of HMAs (decitabine or azacitidine) are available for the treatment of MDS^5^. Several previous studies reported the efficacy of HMAs in different clinical settings. In higher-risk MDS, a 7-day azacitidine regimen (75 mg/m ^2^ daily every 4 weeks) was reported to result in higher response rates and better overall survival (OS) outcomes than treatment with supportive care^6,7^. Decitabine (45 mg/m ^2^ daily for 3 days every 6 weeks) also demonstrated higher response rates, but no apparent survival benefits were found^8,9^. A prospective multicentre phase 2 trial showed the favourable efficacy (32% of overall response and 51% of overall improvement) and tolerable toxicity of a 5-day decitabine regimen (20 mg/m ^2^ daily every 4 weeks) in this group of patients^10^. HMA treatment also showed considerable clinical efficacy in patients with lower-risk MDS^11^. Several prospective trials showed responses of approximately 50–60%, with acceptable toxicities, for a 5-day decitabine regimen (20 mg/m ^2^ daily every 4 weeks)^12,13^. Seven-day azacitidine (75 mg/m ^2^ daily every 4 weeks) was still active in the patients with lower-risk MDS, with an overall response rate of 40–50%^14–16^.

Several studies compared the efficacy of two HMAs in patients with MDS^17,18^. However, these studies were retrospective and therefore definitive conclusions could not be drawn. Moreover, the outcomes of studies in patients with lower-risk MDS seem to be more controversial. For example, a retrospective multicentre study conducted prognostic factor analysis that included the type of HMA; no significant association was found on treatment response and survival outcomes between decitabine and azacitidine^19^. However, another retrospective analysis found better survival outcomes in patients with lower-risk MDS treated with a 5-day decitabine regimen than in those treated with a 7-day azacitidine regimen, although these results also did not reach statistical significance^20^. Recently, a randomised phase 2 study demonstrated that low-dose decitabine therapy (20 mg/m^2^ daily for 3 days, intravenous [IV]) resulted in better response rates than low-dose azacitidine therapy (75 mg/m^2^ daily for 3 days, IV) in patients with lower-risk MDS, especially in those with a poor prognostic feature, i.e., ≥5% bone marrow (BM) blasts^21^. Based on these results, we believe it is necessary to compare the efficacy of HMAs in more widely used regimens, which includes 5 days of decitabine (20 mg/m^2^ daily every 4 weeks) and 7 days of azacitidine (75 mg/m^2^ daily every 4 weeks) in lower-risk MDS patients with poor prognostic features. The hypothesis of this study is that 5 days of decitabine might have greater potential benefits in lower-risk patients with poor prognostic factors than 7 days of azacitidine.

## Methods

### Study design

**Figure 1:**
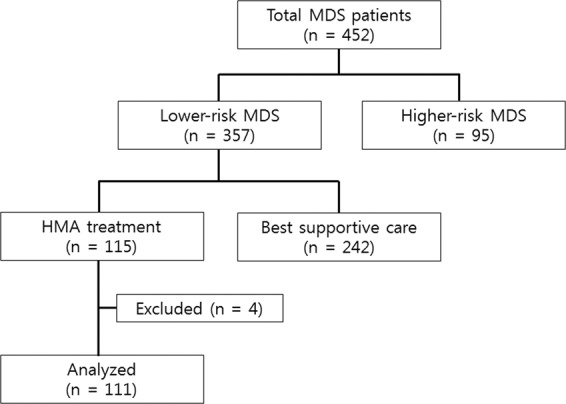
Flow diagram of patients from the Korea University MDS registry from October 2006 to December 2017.

The Korea University MDS registry is a longitudinal cohort that contains data on 452 patients consecutively diagnosed with MDS at the Korea University Medical Center (Korea University Anam, Guro, and Ansan Hospital) from October 2006 to December 2017. In this cohort, 357 patients were classified as having lower-risk MDS, and 115 of them who had poor prognostic features were treated with an HMA (decitabine or azacitidine). Of these 115 patients, four were excluded because they had started the HMA treatment at other hospitals, and their previous medical records were inaccessible (Fig. 1). We retrospectively analysed the data from these 111 patients with low-risk (n = 9) and intermediate 1-risk (n = 102) MDS (based on the IPSS classification).

All methods were carried out in accordance with relevant guidelines and regulations. This study was approved by the institutional review board of Korea University Medical Center with a waiver of informed consent for the collection and analysis of retrospective data.

### Treatment and response evaluation

HMA treatment was initiated in lower-risk MDS patients with poor prognostic features, such as cytopenia, a high percentage of BM blasts, and poor cytogenetics. Decitabine was administered at 20 mg/m^2^ over 1 h daily for 5 days, whereas azacitidine was administered at 75 mg/m^2^ over 30 min daily for 7 days, both intravenously, and the regimens were repeated every 4 weeks. Dose reductions were determined based on the institutional guidelines and physicians’ decisions. Treatments were continued on the basis of the clinical responses and conditions of the patients. Initial BM aspiration and biopsy were performed at the end of 2–6 cycles of HMA treatments. Transfusion dependence was defined on the basis of the requirement for more than 4 units of red blood cells (RBCs) or 16 units of platelets (PLTs) in the 8 week period before treatment.

Treatment responses were assessed according to the modified 2006 International Working Group (IWG) response criteria^22^ and were evaluated in patients who received at least one cycle of HMA therapy. The overall response rate (ORR) included complete remission (CR), partial remission (PR), marrow CR (mCR), and haematologic improvement (HI). HI included at least one of the following: erythroid response (HI-E), PLT response (HI-P), or neutrophil response (HI-N). The RBC and PLT transfusion responses were defined as a reduction of at least 4 units of RBC transfusions and at least 16 units of PLT transfusions, respectively, in 8 weeks compared to the number of transfusions in the 8 weeks before treatment. Survival outcomes were also assessed using the modified 2006 IWG response criteria. OS was defined as the time between the start of treatment and death from any cause. Event-free survival (EFS) was measured from the time of treatment initiation until treatment failure or death from any cause. Progression-free survival (PFS) was defined as the time from the start of treatment to disease progression or death from MDS. Adverse events were evaluated using the Common Terminology Criteria for Adverse Events version 4.0.

### Statistical analysis

Categorical variables were evaluated using the Chi-squared test or Fisher’s exact test. Continuous variables were evaluated using Student’s *t*-test. Backward stepwise logistic regression analysis was used to estimate the association between ORR, HMAs, and other prognostic factors. Survival outcomes were calculated using the Kaplan–Meier method and were compared using the log-rank test. Cox’s proportional hazard model with the backward stepwise elimination method was used to analyse the association between survival rates, HMAs and other prognostic factors. All analyses were performed using SPSS statistics version 25.0 software (IBM Corporation, New York, USA) and R software (version 3.5.2). The meta-analysis was performed using the R package “meta.”

## Results

### Baseline characteristics

Patient characteristics are summarized in Table 1. No significant difference was seen in the median ages of patients treated with decitabine (63; range, 20–85 years) and azacitidine (69; range, 30–82 years) (*P* = 0.268). MDS with multilineage dysplasia according to the World Health Organization classification was the most common subtype (n = 56; 50.5%). Sixty patients (54.1%) had RBC-transfusion dependency, and 56 (50.5%) had PLT-transfusion dependency. Most patients had <5% BM blasts (n = 90; 81.1%) and good cytogenetic risk according to the Revised-IPSS (IPSS-R) classification (n = 94; 84.7%)^23^. Based on the IPSS and IPSS-R classifications, 102 (91.9%) and 51 (45.9%) patients were classified as having intermediate-1 risk and intermediate risk, respectively. The risk groups based on the MD Anderson Lower Risk Prognostic Scoring System (LR-PSS)^24^ included category 1 (n = 8; 7.2%), category 2 (n = 66; 59.5%), and category 3 (n = 37; 33.3%). The baseline patient characteristics were well balanced between the decitabine and azacitidine groups and no significant differences were observed between the groups.

**Table 1:** Patient characteristics.

	Total, n (%)	Decitabine, n (%)	Azacitidine, n (%)	*P*
	(n = 111)	(n = 61)	(n = 50)	
Age, median years (range)	66 (20–85)	63 (20–85)	69 (30–82)	0.268
**Sex**				
Male	71 (64.0)	43 (70.5)	28 (56.0)	0.114
Female	40 (36.0)	18 (29.5)	22 (44.0)	
**ECOG performance**				
0–1	105 (94.6)	57 (93.4)	48 (96.0)	0.688
2–3	6 (5.4)	4 (6.6)	2 (4.0)	
**WHO subtypes**				
MDS-SLD	7 (6.3)	3 (4.9)	4 (8.0)	0.181
MDS-MLD	56 (50.5)	28 (45.9)	28 (56.0)	
MDS-RS	2 (1.8)	0	2 (4.0)	
MDS-EB	33 (29.7)	21 (34.4)	12 (24.0)	
MDS-U	12 (10.8)	9 (14.8)	3 (6.0)	
MDS with isolated del 5q	1 (0.9)	0	1 (2.0)	
**Hb, g/dL**				
≥10	13 (11.7)	6 (9.8)	7 (14.0)	0.746
8 to <10	43 (38.7)	25 (41.0)	18 (36.0)	
< 8	55 (49.5)	30 (49.2)	25 (50.0)	
**ANC, ×10**^**9**^**/L**				
≥0.8	72 (64.9)	40 (65.6)	32 (64.0)	0.863
<0.8	39 (35.1)	21 (34.4)	18 (36.0)	
**PLT, ×10**^**9**^**/L**				
≥100	25 (22.5)	11 (18.0)	14 (28.0)	0.216
50 to <100	34 (30.6)	17 (27.9)	17 (34.0)	
<50	52 (46.8)	33 (54.1)	19 (38.0)	
**Transfusion dependence**				
RBC	60 (54.1)	36 (59.0)	24 (48.0)	0.247
PLT	56 (50.5)	35 (57.4)	21 (42.0)	0.107
**BM blasts, %**				
<5	90 (81.1)	46 (75.4)	44 (88.0)	0.092
≥5	21 (18.9)	15 (24.6)	6 (12.0)	
**Cytogenetics (IPSS-R)**				
Very good	2 (1.8)	2 (3.3)	0	0.215
Good	94 (84.7)	53 (86.9)	41 (82.0)	
Intermediate	15 (13.5)	6 (9.8)	9 (18.0)	
Poor	0	0	0	
Very poor	0	0	0	
**IPSS risk group**				
Low	9 (8.1)	3 (4.9)	6 (12.0)	0.295
Intermediate-1	102 (91.9)	58 (95.1)	44 (88.0)	
**IPSS-R risk group**				
Very low	2 (1.8)	2 (3.3)	0	0.178
Low	35 (31.5)	16 (26.2)	19 (38.0)	
Intermediate	51 (45.9)	27 (44.3)	24 (48.0)	
High	23 (20.7)	16 (26.2)	7 (14.0)	
Very high	0	0	0	
**LR-PSS risk group**				
Category 1	8 (7.2)	3 (4.9)	5 (10.0)	0.134
Category 2	66 (59.5)	33 (54.1)	33 (66.0)	
Category 3	37 (33.3)	25 (41.0)	12 (24.0)	

Abbreviations: ECOG: Eastern Cooperative Oncology Group; WHO: World Health Organization; MDS: myelodysplastic syndrome; MDS-SLD: MDS with single lineage dysplasia; MDS-MLD: MDS with multilineage dysplasia; MDS-RS: MDS with ring sideroblasts; MDS-EB: MDS with excess blasts; MDS-U: MDS, unclassified; Hb: haemoglobin; ANC: absolute neutrophil count; PLT: platelet; RBC: red blood cell; BM: bone marrow; IPSS: international prognostic scoring system; IPSS-R: revised-international prognostic scoring system; LR-PSS: lower-risk prognostic scoring system.

### Treatment response

Patients received a median of 5 (range, 1–61) cycles of decitabine and 4 (range, 1–18) cycles of azacitidine treatment. The median number of cycles at response assessment was 4 (range, 1–8) and 4 (range, 1–6) in patients treated with decitabine and azacitidine, respectively. The CR rates were 16.4% (10/61) in the decitabine group and 6.0% (3/50) in the azacitidine group, with borderline significance (*P* = 0.090; Table 2). The ORR in patients treated with decitabine (67.2%, 41/61) was significantly higher than that in patients treated with azacitidine (44.0%, 22/50) (*P* = 0.014). The cytogenetic response rates, including CR and PR, were 50.0% (3/6) and 16.7% (1/6) for decitabine and azacitidine, respectively (*P* = 0.545). The HI-E rates were significantly higher in patients treated with decitabine (68.3% vs 44.2%; *P* = 0.014), whereas no significant difference was observed in the HI-P (*P* = 0.473) and HI-N (*P* = 0.264) rates. The RBC transfusion response rates were 50.0% (18/36) and 29.2% (7/24) in patients treated with decitabine and azacitidine, respectively (*P* = 0.109).

**Table 2:** Treatment responses.

	Total, n (%)	Decitabine, n (%)	Azacitidine, n (%)	*P*
	(n = 111)	(n = 61)	(n = 50)	
CR	13 (11.7)	10 (16.4)	3 (6.0)	0.09
mCR	5 (4.5)	3 (4.9)	2 (4.0)	1
PR	0	0	0	
HI (without CR, mCR, PR)	45 (40.5)	28 (45.9)	17 (34.0)	0.204
SD	26 (23.4)	10 (16.4)	16 (32.0)	0.053
Failure	18 (16.2)	8 (13.1)	10 (20.0)	0.328
Not assessed	4 (3.6)	2 (3.3)	2 (4.0)	0.839
ORR (CR + mCR + PR + HI)	63 (56.8)	41 (67.2)	22 (44.0)	0.014
Cytogenetic response, n	12	6	6	
CR + PR	4 (33.3)	3 (50.0)	1 (16.7)	0.545
**HI**				
HI-E (n = 103)	60 (58.3)	41 (68.3)	19 (44.2)	0.014
HI-P (n = 85)	28 (32.9)	18 (36.0)	10 (28.6)	0.473
HI-N (n = 50)	13 (26.0)	9 (32.1)	4 (18.2)	0.264
**Transfusion response**				
RBC (n = 60)	25 (41.7)	18 (50.0)	7 (29.2)	0.109
PLT (n = 56)	25 (44.6)	16 (45.7)	9 (42.9)	0.835

Abbreviations: CR: complete remission; mCR: marrow CR; PR: partial remission; HI: haematologic improvement; SD: stable disease; ORR: overall response rate; HI-E: HI-erythroid; HI-P: HI-platelet; HI-N: HI-neutrophil; RBC: red blood cell; PLT: platelet.

In the univariate analysis for ORR following decitabine vs azacitidine treatment (67.2% vs 44.0%; hazards ratio [HR], 2.609; 95% confidence interval [CI], 1.204–5.652; *P* = 0.015), a haemoglobin (Hb) concentration <8 g/dL (69.1% vs 44.6%; HR, 2.772; 95% CI, 1.274–6.032; *P* = 0.010) and ≥5% BM blasts (81.0% vs 51.1%; HR, 4.065; 95% CI, 1.268–13.032; *P* = 0.018) were significantly associated with a higher ORR. Other risk factors showed no significant associations. In the multivariate analysis using the backward stepwise elimination method including the types of HMA, age, sex, Hb level, absolute neutrophil count (ANC), PLT count, RBC- and PLT-transfusion dependency, BM blasts, cytogenetics, and risk groups based on IPSS-R and LR-PSS, treatment with decitabine (HR, 2.553; 95% CI, 1.116–5.840; *P* = 0.026), Hb concentration of <8 g/dL (HR, 3.073; 95% CI, 1.340–7.048; *P* = 0.008), and ≥5% BM blasts (HR, 3.739; 95% CI, 1.102–12.683; *P* = 0.034) were all significantly associated with higher ORR. However, no significant associations were observed for the other prognostic factors.

### Survival analysis

**Figure 2:**
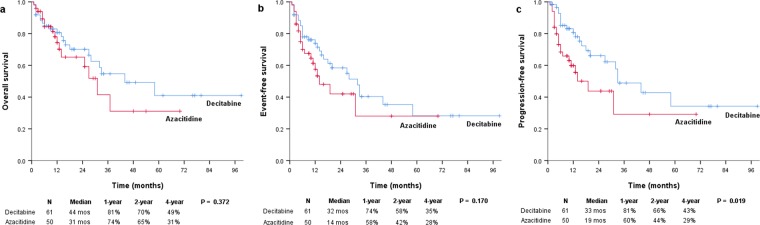
Kaplan–Meier survival analysis. (**a**) The median OS was 44 months for decitabine and 31 months for azacitidine (*P* = 0.372). (**b**) The median event-free survival was 32 and 14 months for decitabine and azacitidine, respectively (*P* = 0.170). (**c**) The median PFS was significantly prolonged following decitabine treatment compared to azacitidine treatment (33 vs 19 months; *P* = 0.019).

**Figure 3:**
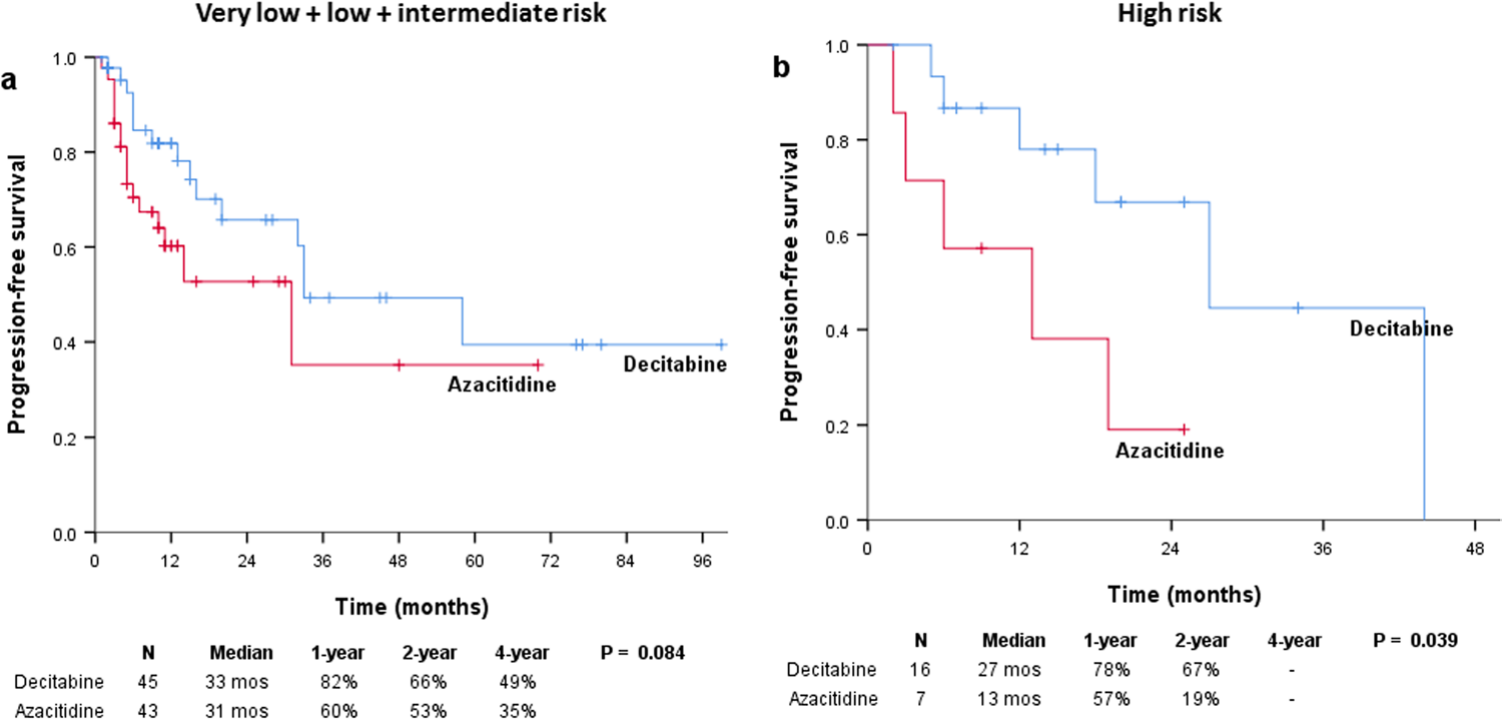
Kaplan–Meier PFS curves in the two risk subgroups by IPSS-R. (**a**) The median PFS was 33 months for decitabine and 31 months for azacitidine in the very low to intermediate risk subgroup (*P* = 0.084). (**b**) The median PFS was 27 and 13 months in the decitabine and azacitidine groups, respectively in the high risk subgroup (*P* = 0.039).

The median follow-up duration was 15 (range, 1–99) and 10.5 (range, 1–70) months in the decitabine (n = 61) and azacitidine (n = 50) groups, respectively. The median OS was 44 and 31 months in the decitabine and azacitidine groups, respectively (*P* = 0.372; Fig. 2a). In the decitabine and azacitidine groups, the 1-year OS rates were 81% and 74%, respectively; the 4-year OS rates were 49% and 31%, respectively. The median EFS was 32 and 14 months for patients treated with decitabine and azacitidine, respectively (*P* = 0.170; Fig. 2b). The median PFS was significantly prolonged in patients treated with decitabine than in those treated with azacitidine (33 vs 19 months; *P* = 0.019; Fig. 2c). In the decitabine and azacitidine groups, the 1-year PFS rates were 81% and 60%, respectively; the 2-year PFS rates were 66% and 44%, respectively; and the 4-year PFS rates were 43% and 29%, respectively. The Kaplan-Meier survival analysis of the risk groups based on the IPSS-R showed that PFS was better in the very low to intermediate risk subgroup treated with decitabine than in those treated with azacitidine, with borderline significance (*P* = 0.084; Fig. 3a). PFS was significantly longer in the high risk subgroup who received decitabine compared to azacitidine (*P* = 0.039; Fig. 3b).

To analyse the significance of the difference in PFS in response to specific HMAs, we performed univariate and multivariate analyses including other prognostic factors. In the univariate analysis, treatment with decitabine (HR, 0.489; 95% CI, 0.264–0.907; *P* = 0.023) and achievement of CR (HR, 0.133; 95% CI, 0.018–0.964; *P* = 0.046) were significant favourable prognostic factors, whereas a poor cytogenetic risk based on IPSS (HR, 2.555; 95% CI, 1.225–5.326; *P* = 0.012) was a significant unfavourable prognostic factor (Table 3). In the multivariate analysis using the backward stepwise elimination method including types of HMA, age, sex, Hb level, ANC, PLT count, RBC- and PLT-transfusion dependency, BM blasts, cytogenetics, and risk groups based on the IPSS-R and LR-PSS, we found that treatment with decitabine (HR, 0.496; 95% CI, 0.257–0.957; *P* = 0.037) and achievement of CR (HR, 0.122; 95% CI, 0.015–0.993; *P* = 0.049) were significant prognostic factors for better survival, whereas ANC below 0.8 × 10^9^/L (HR, 1.905; 95% CI, 1.032–3.515; *P* = 0.039) was a significant poor prognostic factor. Poor cytogenetic risk (HR, 2.136; 95% CI, 0.992–4.556; *P* = 0.052) also unfavourably affected survival with borderline significance. The other prognostic factors showed no significant associations with better survival.

**Table 3:** Prognostic factor analysis for progression-free survival.

	Univariate			Multivariate		
	HR	95% CI	*P*	HR	95% CI	*P*
**HMA**						
Azacitidine	1			1		
Decitabine	0.489	0.264–0.907	0.023	0.496	0.257–0.957	0.037
**Age, years**						
<65	1					
≥65	1.236	0.676–2.259	0.491			
**Sex**						
Male	1					
Female	0.824	0.434–1.567	0.556			
**Hb, g/dL**						
≥8	1					
<8	1.091	0.598–1.990	0.777			
**ANC, ×10**^**9**^**/L**						
≥0.8	1			1		
<0.8	1.623	0.889–2.961	0.115	1.905	1.032–3.515	0.039
**PLT, ×10**^**9**^**/L**						
≥50	1					
<50	1.077	0.588–1.972	0.81			.
**Transfusion (RBC)**						
Independent	1					
Dependent	1.061	0.580–1.942	0.848			
**Transfusion (PLT)**						
Independent	1			1		
Dependent	1.514	0.830–2.763	0.177	1.658	0.860–3.196	0.131
**BM blasts, %**						
<5	1			1		
≥5	0.715	0.302–1.696	0.447	2.203	0.839–5.784	0.109
**Cytogenetics (IPSS)**						
Good + intermediate	1			1		
Poor	2.555	1.225–5.326	0.012	2.126	0.992–4.556	0.052
**IPSS risk**						
Low	1					
Intermediate-1	0.79	0.281–2.222	0.655			
**IPSS-R risk**						
Very low + low	1					
Intermediate + high	1.255	0.662–2.380	0.487			
**LR-PSS risk**						
Category 1–2	1					
Category 3	1.142	0.594–2.195	0.691			
**Response**						
Others	1			1		
CR	0.133	0.018–0.964	0.046	0.122	0.015–0.993	0.049

Abbreviations: HR: hazard ratio; CI: confidence interval; HMA: hypomethylating agent; Hb: haemoglobin; ANC: absolute neutrophil count; PLT: platelet; RBC: red blood cell; BM: bone marrow; IPSS: international prognostic scoring system; IPSS-R: revised-international prognostic scoring system; LR-PSS: lower-risk prognostic scoring system; CR: complete remission.

### Meta-analysis

**Figure 4:**
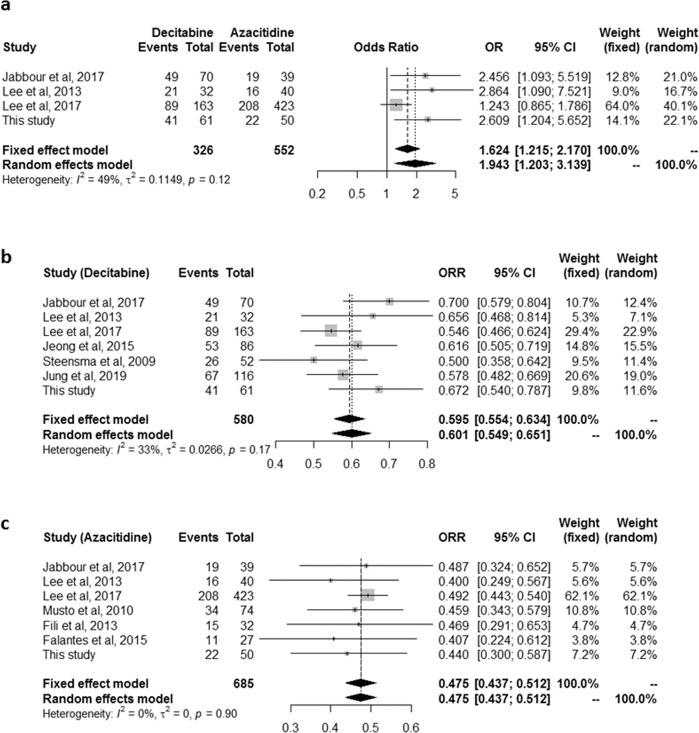
Meta-analysis and forest plots of ORR in patients treated with decitabine and azacitidine. (**a**) The data from four studies (878 patients; 326 treated with decitabine and 552 treated with azacitidine) were analysed to compare ORR between decitabine and azacitidine treatment. Cochran’s Q value (*P* = 0.119) and the I^2^ value (48.8%) indicate moderate heterogeneity among the four studies. Decitabine treatment showed significantly better ORR than azacitidine treatment (odds ratio, 1.943; 95% CI, 1.203–3.139; P = 0.007; random effect model). (**b**) A total of 580 patients treated with decitabine were analysed. Cochran’s Q test value of *P* = 0.17 and the I^2^ value of 33% indicated low heterogeneity among the seven studies. Decitabine had an estimated pooled ORR of 59.5% based on a fixed effects model. (**c**) A total of 685 patients treated with azacitidine were analysed. Cochran’s Q test value of *P* = 0.90 and the I^2^ value of 0% indicated low heterogeneity among the seven studies. Azacitidine had an estimated pooled ORR of 47.5% based on a fixed effects model.

We performed a meta-analysis using previously published data and data from our patient cohort with lower-risk MDS. For the comparison of ORR between decitabine and azacitidine, one randomised study^21^, two retrospective studies^19,20^, and our study were included (Fig. 4a). The data from these four studies (878 patients; 326 treated with decitabine and 552 treated with azacitidine) were available. Cochran’s Q test for heterogeneity yielded a *P-*value of 0.119 and I^2^ = 48.8%, indicating moderate heterogeneity among the four studies. Decitabine treatment was found to be significantly advantageous over azacitidine treatment in terms of ORR (odds ratio, 1.943; 95% CI, 1.203–3.139; *P* = 0.007; four studies, random effect model). The sensitivity analysis of the above studies excluding our study also showed that ORR was significantly higher in patients treated with decitabine compared to azacitidine (odds ratio, 1.809; 95% CI, 1.023–3.199; *P* = 0.042; three studies, random effect model). In the analysis of ORR for decitabine treatment, one randomised study^21^, three non-randomised prospective studies^10,12,13^, two retrospective studies^19,20^ providing available information on lower-risk MDS (low or intermediate-1 based on IPSS), and our study were included (Fig. 4b). In total, 580 patients were examined; a Cochran’s Q test value of *P = *0.17 and I^2^ = 33% indicated low heterogeneity among the seven studies. Decitabine had an estimated pooled ORR of 59.5% based on a fixed effects model. In the analysis of ORR for azacitidine, one randomised study^21^, one non-randomised prospective study^15^, four retrospective studies^14,16,19,20^ providing available information on lower-risk MDS, and our study were included (Fig. 4c). Overall, 685 patients were examined; a Cochran’s Q test value of *P = *0.90 and I^2^ = 0% indicated low heterogeneity among the seven studies. Azacitidine treatment had an estimated pooled ORR of 47.5% (95% CI, 43.7–51.2%) based on a fixed effects model. The results of the pooled analyses indicated that decitabine treatment had a significantly higher ORR than azacitidine treatment (59.5% [95% CI, 55.4–63.4%] vs 47.5% [95% CI, 43.7–51.2%]; *P* < 0.001) and these results were comparable to our results (67.2% vs 44.0%; *P* = 0.014).

### Causes of death

The causes of deaths have been summarized in Table 4. Among the patients treated with decitabine, 14 (66.7%) died from MDS-related causes, of which infection (n = 8) was the most common followed by disease progression (n = 4). Seven patients died due to MDS-unrelated causes, such as solid cancer (n = 3), myocardial infarction (n = 1), interstitial lung disease (n = 2), and epilepsy (n = 1). Among the patients who received azacitidine, 15 (93.8%) died from MDS-related causes. Infection (n = 7) was again the most frequent cause of MDS-related death, followed by disease progression (n = 6). One patient succumbed to solid cancer, an MDS-unrelated cause. No significant differences were noted between the incidence of MDS-related and MDS-unrelated deaths (*P* = 0.104).

**Table 4:** Causes of death.

Causes of death	Total, n (%)	Decitabine, n (%)	Azacitidine, n (%)	*P*
	(n = 37)	(n = 21)	(n = 16)	
MDS-related death	29 (78.4)	14 (66.7)	15 (93.8)	0.104
Disease progression	10	4	6	
Infection	15	8	7	
Bleeding	4	2	2	
MDS-unrelated death	8 (21.6)	7 (33.3)	1 (6.2)	
Solid cancer	4	3	1	
Myocardial infarction	1	1	0	
Interstitial lung disease	2	2	0	
Epilepsy	1	1	0	

### Safety

Table 5 summarizes the haematological adverse events following treatments with decitabine and azacitidine. These included grade 3 or higher anaemia in 18 (16.2%), neutropenia in 35 (31.5%), thrombocytopenia in 30 (27.0%), and febrile neutropenia in 29 (26.1%) patients. No significant differences in the incidence of adverse events were observed following treatment with decitabine and azacitidine.

**Table 5:** Toxicity analysis.

Haematologic adverse events	Total, n (%)	Decitabine, n (%)	Azacitidine, n (%)	*P*
(Grade 3 or higher)	(n = 111)	(n = 61)	(n = 50)	
Haemoglobin	18 (16.2)	11 (18.0)	7 (14.0)	0.566
Neutrophils	35 (31.5)	21 (34.4)	14 (28.0)	0.468
Platelets	30 (27.0)	17 (27.9)	13 (26.0)	0.825
Febrile neutropenia	29 (26.1)	18 (29.5)	11 (22.0)	0.37

## Discussion

In our retrospective cohort analysis of lower-risk MDS patients with poor prognostic features, higher ORR and HI-E rates were observed after treatment with a 5-day decitabine regimen (20 mg/m^2^ daily every 4 weeks) than after treatment with a 7-day azacitidine regimen (75 mg/m^2^ daily every 4 weeks) (ORR, 67.2% vs 44.0%; *P* = 0.014 and HI-E, 68.3% vs 44.2%; *P* = 0.014). The groups treated with the two HMAs showed no significant differences with respect to OS or EFS. Interestingly, PFS was higher in the 5-day decitabine regimen group than in the 7-day azacitidine regimen group (median, 33 vs 19 months; *P* = 0.019).

The benefit of HMAs in patients with lower risk has also been addressed in several previous studies. One randomised study reported that a 3-day low-dose decitabine regimen (20 mg/m^2^ SC per day for 3 consecutive days every 28 days) showed a protocol-defined overall response rate of 23.3%, including CR, mCR, PR, and HI, which was similar to the 22.7% response rate to a weekly decitabine regimen (20 mg/m^2^ SC per day once every 7 days every 28 days)^11^. A prospective phase 2 study suggested promising clinical efficacy (CR, 19%; HI, 38%; ORR, 58%) for low-dose azacitidine (75 mg/m^2^/d SC for 5 days every 28 days) in lower-risk MDS patients^14^. In addition, a recent randomised study demonstrated better outcomes (ORR, 70% vs 49%; *P* = 0.03) for low-dose decitabine (20 mg/m^2^ daily for 3 days every 4 weeks) than for azacitidine (75 mg/m^2^ daily for 3 days every 4 weeks) in patients with lower-risk MDS^21^. However, only limited information is available regarding the clinical efficacy and safety of 5-day decitabine (20 mg/m^2^ daily for every 4 weeks) and 7-day azacitidine (75 mg/m^2^ daily every 4 weeks) regimens in lower-risk MDS, which are the most widely used regimens in clinical practice. In the present study, we observed an ORR of 67.2% in patients treated with a 5-day decitabine regimen (20 mg/m^2^ daily every 4 weeks), 44.0% in those treated with a 7-day azacitidine regimen (75 mg/m^2^ daily every 4 weeks), and 56.8% in all lower-risk patients with poor prognostic features. The median OS was 44 months in patients treated with a 5-day decitabine regimen, 31 months in those treated with a 7-day azacitidine regimen, and 37 months in all patients. Considering the patients in this study had at least one adverse prognostic feature despite being categorised as lower-risk based on IPSS, the similar efficacy in this study compared with that reported in previous studies might be suggestive of the potential benefit of a 5-day decitabine regimen in this group of patients.

Regarding toxicity, grade 3 or higher neutropenia and thrombocytopenia occurred in 34.4% and 27.9% of decitabine-treated patients, respectively, in this study. In the azacitidine-treated group, grade 3 or higher thrombocytopenia developed in 12.5% of patients. Overall, the development of toxicity seems to be higher than that previously reported^11,14^. This study is based on a retrospective cohort enrolling consecutive unselected patients in the real world. Therefore, more fragile patients might be included compared with previous studies. Second, we analysed data from patients with lower-risk MDS who had poor prognostic features, which included transfusion dependency, cytopenia, high percentage of BM blasts, and poor cytogenetics. These features might partially contribute to the higher development of toxicity for both decitabine and azacitidine.

A previous meta-analysis suggested that compared with conventional care, both decitabine and azacitidine might be active in both the response and HI for treating MDS^25^. However, which of the two drugs is superior with respect to survival seems to depend on the clinical situation. For example, compared with supportive care, azacitidine showed a survival benefit in patients with higher-risk MDS, but decitabine failed to show a survival benefit^25^. Currently, there is limited prospective comparison between the two drugs with respect to survival. Three retrospective studies performed a direct comparison of 5-day decitabine and 7-day azacitidine regimens;^17,18,20^ however, no significant differences were observed in survival outcomes. If patients are narrowed down to low-risk patients, the situation might be a little different. Some previous studies suggested that decitabine is superior in this group of patients. Recent prospective studies reported that the ORRs of patients with lower-risk MDS to decitabine were approximately 60%^12,13^. Overall responses to azacitidine in lower-risk MDS were reported to be approximately 40% in this group of patients^14–16^. The results of this study are also consistent with this trend. In this study, decitabine demonstrated a significantly higher ORR (HR, 2.553; 95% CI, 1.116–5.840; *P* = 0.026) and HI-E (68.3% vs 44.2%; *P* = 0.014) compared with azacitidine, and treatment with decitabine was a significant prognostic factor for higher ORR in the multivariate analysis. The results of the meta-analysis in this study strengthen these outcomes, which demonstrated that patients who received decitabine treatment had significantly better ORR than those who received azacitidine (odds ratio, 1.943; 95% CI, 1.203–3.139; *P* = 0.007). The pooled analyses also showed that decitabine might be significantly more beneficial than azacitidine with respect to ORR (59.5% vs 47.5%; *P* < 0.001). OS and EFS were comparable in the decitabine- and azacitidine-treated groups, and this finding was consistent with that of previous studies comparing these two HMAs^17–20^.

In this study, compared with azacitidine, decitabine demonstrated a significantly higher median PFS, and treatment with decitabine was significantly associated with favourable PFS in the multivariate analysis. This result was an outcome that was not observed in previous studies. There are some possible contributory factors to consider. Although not statistically significant, more patients who received azacitidine experienced treatment failure, including disease progression, than those who received decitabine (20.0% vs 13.1%; *P* = 0.328) (Table 2), and more patients treated with decitabine died from MDS-unrelated causes than those treated with azacitidine (33.3% vs 6.2%; *P* = 0.104) (Table 4). Considering that most MDS-unrelated causes of death included various underlying diseases, such as solid cancer, myocardial infarction, and interstitial lung disease, it is likely that patients treated with decitabine might have had more comorbidities than those treated with azacitidine. Additionally, heterogeneity in salvage therapy after the failure of HMA therapy between the two groups could have influenced the discrepancy between OS and PFS. Of the 43 patients who received salvage therapy, allogenic stem cell transplantation (allo-SCT) was performed in 10 patients in the decitabine group and four in the azacitidine group (data not shown). The higher percentage of salvage allo-SCT in the decitabine group might have affected PFS more favourably.

Another interesting point is that the statistical power was reduced for detecting the PFS improvement owing to decitabine over azacitidine in the very low-to-intermediate risk group in subgroup analyses according to IPSS-R (Fig. 3a); in contrast, the PFS benefit from the 5-day decitabine regimen was maintained in the high-risk group according to IPSS-R (Fig. 3b). This finding might support the results of this study, which showed that decitabine may have some additional benefit in a ‘higher-risk’ subset of lower-risk MDS patients and that the treatment regimen of 5 days of decitabine might be more effective in treating lower-risk MDS with poor prognostic features.

Mechanisms that might explain the differences between the clinical effects of decitabine and azacitidine have not yet been elucidated. Specific gene mutations might affect the clinical responses to decitabine or azacitidine therapy. In a previous study, mutations in CBL, IDH2, DNMT3A, ASXL1, and TP53 were associated with poor prognosis in patients with MDS^26^. Among them, TP53 mutation was reported to affect response to decitabine or azacitidine treatment^27–29^. Although several studies have shown an association between TP53 mutations and poor survival outcomes in patients treated with azacitidine^27–29^, some studies have reported an association between TP53 mutations and higher decitabine sensitivity^30^. In addition, compared with azacitidine, decitabine is known to be a more potent hypomethylating agent, and therefore, drug-specific loci or gene-specific DNA methylation might explain the differences in their therapeutic effects^31,32^. However, we could conduct analysis only at the cytogenetic level because genetic mutations related to MDS could not be identified. This is the major limitation of this study.

There are several more limitations in this study. This analysis was based on a retrospective cohort with a relatively small sample size. Hence, any confirmatory conclusions cannot be drawn from the results of this study. Next, we used IPSS for treatment decisions in MDS, and thus, we conducted analyses based on IPSS in this study. The use of IPSS to predict prognosis could result in a heterogeneity in outcomes within the lower-risk groups. Currently, IPSS-R is known to have greater prognostic value than IPSS^23^. In fact, a considerable number of patients in the lower-risk group as categorised by IPSS in this study were re-categorised to the high-risk group by IPSS-R. Therefore, future studies should prospectively enrol lower-risk patients as categorised by IPSS-R.

In conclusion, the results of this study show that 5-day decitabine (20 mg/m^2^ daily every 4 weeks) therapy might have a greater benefit than 7-day azacitidine (75 mg/m^2^ daily every 4 weeks) therapy in patients with lower-risk MDS, especially in those with poor prognostic features. Currently, the consensus regarding HMA treatment in patients with lower-risk MDS is low-dose therapy (3-day decitabine regimen [20 mg/m^2^ daily for 3 days every 4 weeks] or 5-day azacitidine regimen [75 mg/m^2^ daily for 5 days every 4 weeks])^33^. In our opinion, the results of this study support the need for investigating the role of the conventional 5-day decitabine regimen in lower-risk MDS patients with poor prognostic features.

## Data Availability

The datasets generated during and/or analysed during the current study are available from the corresponding author on reasonable request.
